# Distribution and origin of anomalously high permeability zones in Weizhou formation, Weizhou 12-X oilfield, Weixinan Sag, China

**DOI:** 10.1007/s12145-021-00670-x

**Published:** 2021-07-13

**Authors:** Guosong Chen, Yuanlin Meng, Jinlai Huan, Youchun Wang, Lei Zhang, Lihua Xiao

**Affiliations:** 1 School of Geosciences, Northeast Petroleum University, Daqing, 163318 Heilongjiang China; 2 Zhanjiang branch of China National Offshore Oil Corporation (Cnooc), Zhanjiang, 524057 Guangdong China; 3 Exploration and Development Institute of Daqing Oilfield Co Ltd, Daqing, 163318 Heilongjiang China; 4 Institute of Nuclear Industrial Geology, Beijing, 100029 China

**Keywords:** Grain size, Sorting, Diagenesis, Early hydrocarbon emplacement, Weixinan Sag

## Abstract

In order to study the dominant seepage channel of the third member of Weizhou formation (E*w*_3_) in Weizhou 12-X oilfield, Weixinan Sag, Beibu Gulf Basin, and tap the potential of remaining oil. The distribution and causes of the anomalously high permeability zones in Weizhou Formation were studied by using conventional core physical property analysis, scanning electron microscope, laser particle size analysis, X-ray diffraction and thin section microscopic identification. As the results show, vertically,there are three anomalously high permeability zones in the A_1_^1^, A_1_^2^ and A_2_^1^ micro-stage of the middle diagenetic stage, with the depth range of 2300 m ~ 2400 m, 2400 m ~ 2600 m, 2600 m ~ 2900 m respectively. Grain size, sorting, dissolution and early emplacement of hydrocarbons are the main causes of anomalously high permeability zones. Although both grain size and sorting affect porosity and permeability, the effect of grain size on permeability is stronger than sorting, and sorting has a stronger effect on porosity than grain size. Magmatic hydrothermal and organic acid promote dissolution and concomitant porosity and permeability increase by dissolving unstable minerals. The early emplacement of hydrocarbons retard the cementation and accompanying porosity and permeability reduction by reducing the water-rock ratio. Finally, sandstone reservoirs in the E*w*_3_ are characterized by anomalously high permeability zones.

## Introduction

The spatial distribution characteristics of reservoir physical properties in petroliferous basin was controlled by the mutual restriction among heterogeneity, differential diagenesis and fluid activity of clastic reservoir, which has an important influence on the migration and accumulation of oil and gas. The formation heterogeneity controlled by sedimentation and structure can promote the difference of diagenesis of clastic rocks (Luo et al. 2016a). Fluid activity can change the formation heterogeneity and strengthen the difference of diagenesis (Xie and Wang 2003; Li 2016). As a result, the reservoir heterogeneity is strengthened (Luo et al. 2016b; Luo et al. 2020). The concept of anomalously porosity and permeability breaks the traditional viewpoint of “Reservoir physical properties become worse with the increase of depth” (Bloch et al. 2002), and deepens the understanding of the effect of different geological factors on reservoir porosity. Meng (2004) first introduced the concept of anomalously high porosity zones into China. After that, many scholars focused on the distribution, origin and prediction of anomalously high porosity zones (Meng et al. 2006, 2008, 2010; Zhu et al. 2007; Lander and Bonnsell 2010; Ehrenberg et al. 2012; Taylor et al. 2010; Cao et al. 2014; Yuan et al. 2015). As far as oilfield exploration and development are concerned, exploration workers focus on oil and gas reserves and reservoir porosity, while development workers pay more attention to oilfield production and reservoir permeability. Although reservoir porosity and permeability are equally important in the research field of petroleum industry, their research progress is very different. The main reason is that there is no one-to-one correspondence between reservoir porosity and permeability (Carcione et al. 2019), and the research difficulty of permeability is far greater than that of porosity. At present, for the clastic reservoir with less than 10% interstitial material, the prediction error of porosity is less than 2%. But the prediction of permeability is difficult. In generally, according to the correlation between porosity and permeability, the prediction error is large. In offshore oilfield with sparse well pattern, even if the error of logging interpretation permeability is less than one order of magnitude, it is very good (Yuan et al. 2019). With the continuous improvement of exploration and development technology in the oil industry, on the whole, the major oilfields have entered the middle and late stage of development. Especially the development of offshore oil and gas fields with high drilling cost and risk. Unfortunately, due to the economic recovery after the global pandemic of new coronavirus, the global oil price is soaring. In order to increase oil reserves and production and meet the national energy demand, the prediction of seepage channel has become an urgent problem. In particular, there is a lack of systematic research and discussion on the effect of different geological factors on reservoir permeability. This paper attempts to take Weizhou 12-X oilfield in Weixinan Sag as an example, through conventional physical property analysis, scanning electron microscope (SEM), laser particle size analysis, X-ray diffraction and microscopicsection identification (MSI), to study the effect of clastic rock particle structure, diagenesis and fluid activity on reservoir permeability, and to clarify the distribution and origin of anomalously high permeability in the E*w*_3_ of Weixinan Sag. It provides scientific basis for efficient exploration and development of this oilfield and other offshore oil and gas fields.

## Geological background and basic reservoir characteristics

**Fig. 1:**
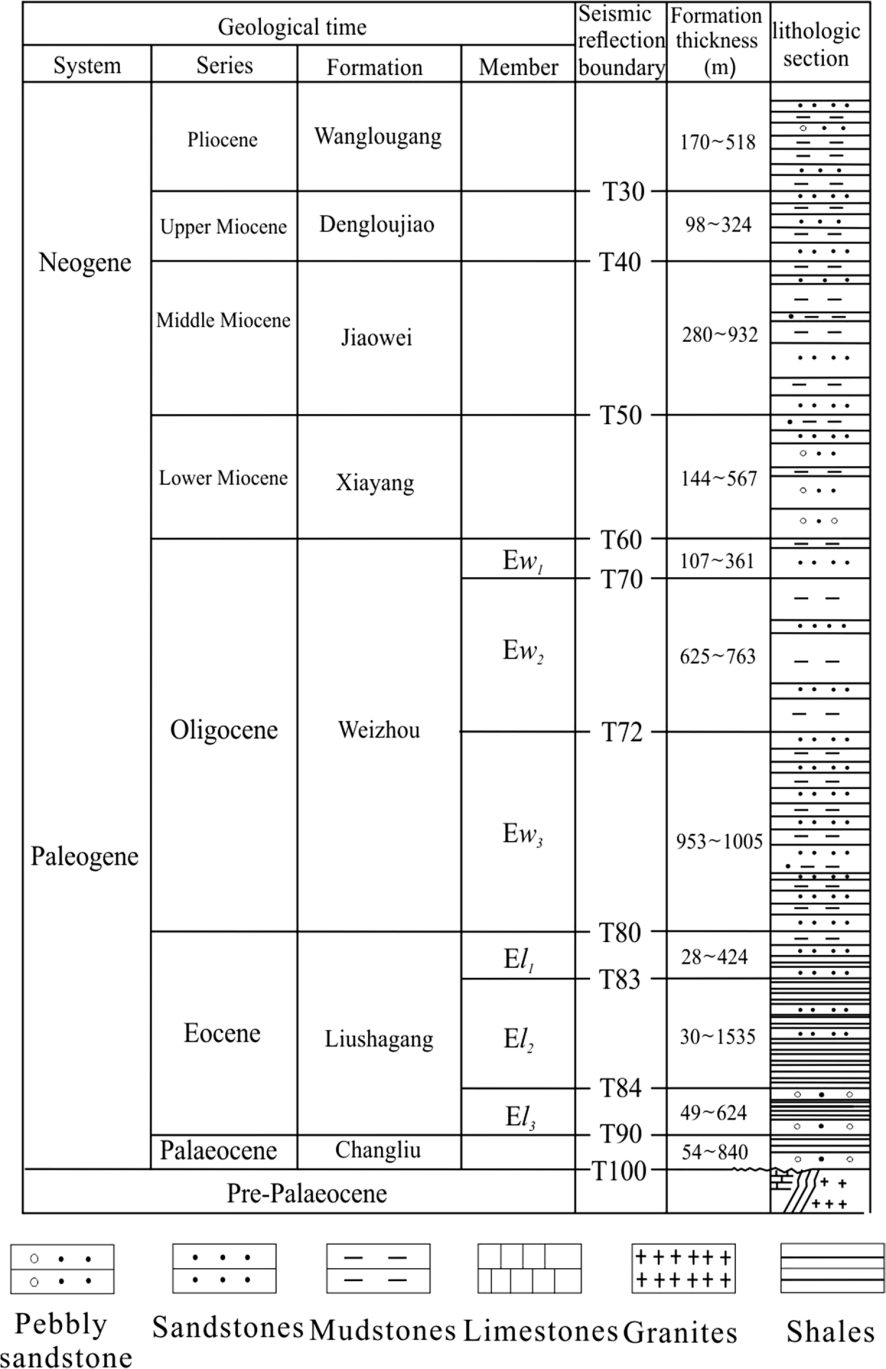
Stratigraphic histogram in the Weixinan Sag (Modified from Chen et al. 2021)

**Fig. 2:**
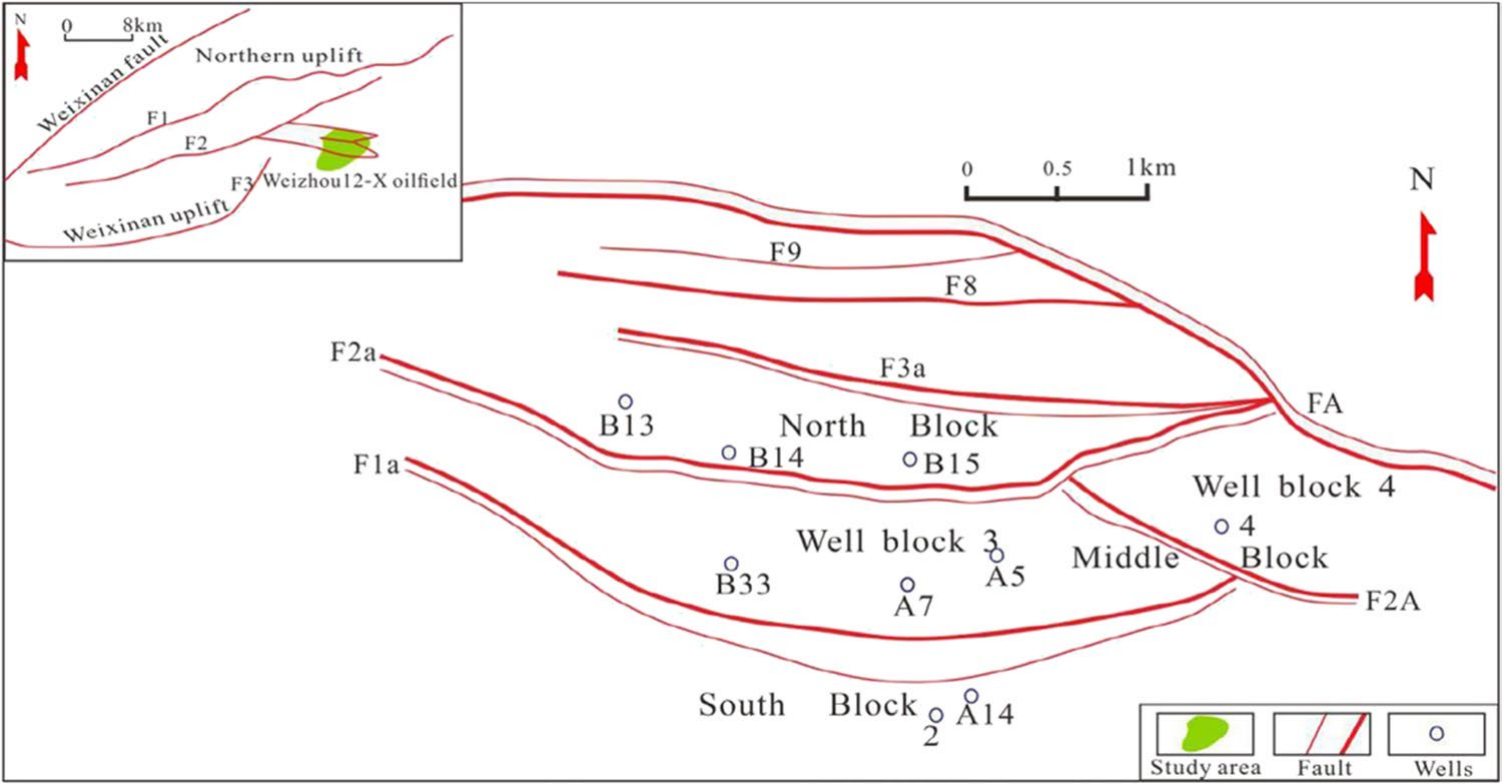
Location of study area and division of tectonic unit

Weixinan Sag is located in Beibu Gulf Basin, China, and occupies about 3800km^2^. Weizhou 12-X oilfield is located in Weixinan Sag. Changliu (E*c*), Liushagang (E*l*), Weizhou (E*w*), Xiayang (N*x*), Jiaowei (N*j*), Dengloujiao (N*d*), Wanglougang (N*w*) and Quaternary (Q) are deposited from bottom to top in the basin (Xu et al. 2020; Chen et al. 2021) (Fig. 1). The Beibu Gulf Basin experienced three periods of rifts in the early Paleocene, late Paleocene and late Eocene, respectively (Guo et al. 2010). Horizontally, Weizhou 12-X oilfield is the main target area of this study, which is composed of North block, Middle block and South block (Fig. 2). The Middle block can be further subdivided into two well blocks: Well Block 3 and Well Block 4 (Fig. 2). In addition, the sedimentary facies of Weizhou Formation is a set of fluvial delta (Chen et al. 2021). Vertically, The third member of Weizhou Formation (E*w*_3_) is the main target of this study, and the E*w*_3_ is the main reservoir, and the second member of Liushagang Formation (E*l*_2_) is the main source rocks. The thick mudstone of shallow lacustrine and semi-deep lacustrine subfacies developed in the E*w*_2_, which is a set of good regional cap rocks, forming a relatively good normal source (E*l*_2_)-reservoir (E*w*_3_)-cap (E*w*_2_) assemblage. The clastic reservoirs in the E*w*_3_ are mainly lithic quartz sandstone and feldspar lithic sandstone, which are mainly fine sandstone and very fine sandstone. The clastic particles are well sorted and rounded, and most of them are sub round to sub prismatic (Xu et al. 2020).

According to the data of *R*_o_, homogenization temperature of fluid inclusion, mudstone pyrolysis, XRD, SEM and thin section identification of clastic rocks in the study area of Weixinan Depression, and referring to the subdivision scheme of diagenetic stage of clastic rocks (Meng et al. 2020), the diagenesis of clastic rocks in Weizhou 12-X oilfield in Weixinan Depression is divided into early diagenetic stage A and B (Table 1), the middle diagenetic stage is further subdivided into A_1_^1^, A_1_^2^, A_2_^1^ and A_2_^2^ diagenetic micro stages, with bottom depths of 1300 m±, 2100 m±, 2400 m±, 2700 m±, 3400 m±, 3900 m±, respectively. In the early diagenetic stage A and B, early compaction and cementation are dominant, and the sandstone is gradually consolidated from the loose state. In the middle diagenetic stage A_1_^1^ micro stage, the kerogen in the E*l*_2_ began to generate hydrocarbon. Organic acids and CO_2_ were generated while oil and gas generated. The organic acids and CO_2_ dissolved in water and formed acidic hot fluid. Under the influence of acidic hot fluid, the dissolution in the E*w*_3_ is obviously enhanced, but the development degree of dissolution is different in each diagenetic micro stages. In the A_1_^1^, A_1_^2^, A_2_^1^ and A_2_^2^ diagenetic micro period, the early relatively weak dissolution facies, the early relatively strong dissolution facies, the middle strong dissolution facies and the late dissolution facies are developed respectively (Table 1).

**Table 1:**
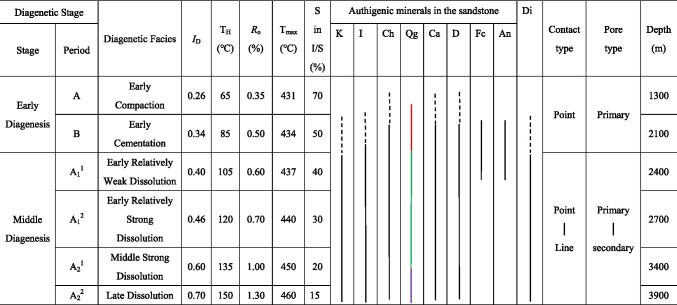
The diagenetic sages and main characteristics in theWeixinan Sag

*I*_D_ Diagenesis Indes, *T*_*H*_ Homogenization Temperature, *K* Kaolinite, *I* Illite, *Ch* Chlorite, *Qg* Quartz Overgrowth, *Ca* Calcite, *D* Dolomite, *Fc* Ferrocalcite, *An* Ankerite, *Di* Dissolution

## Distribution of the anomalously high permeability zones

**Fig. 3:**
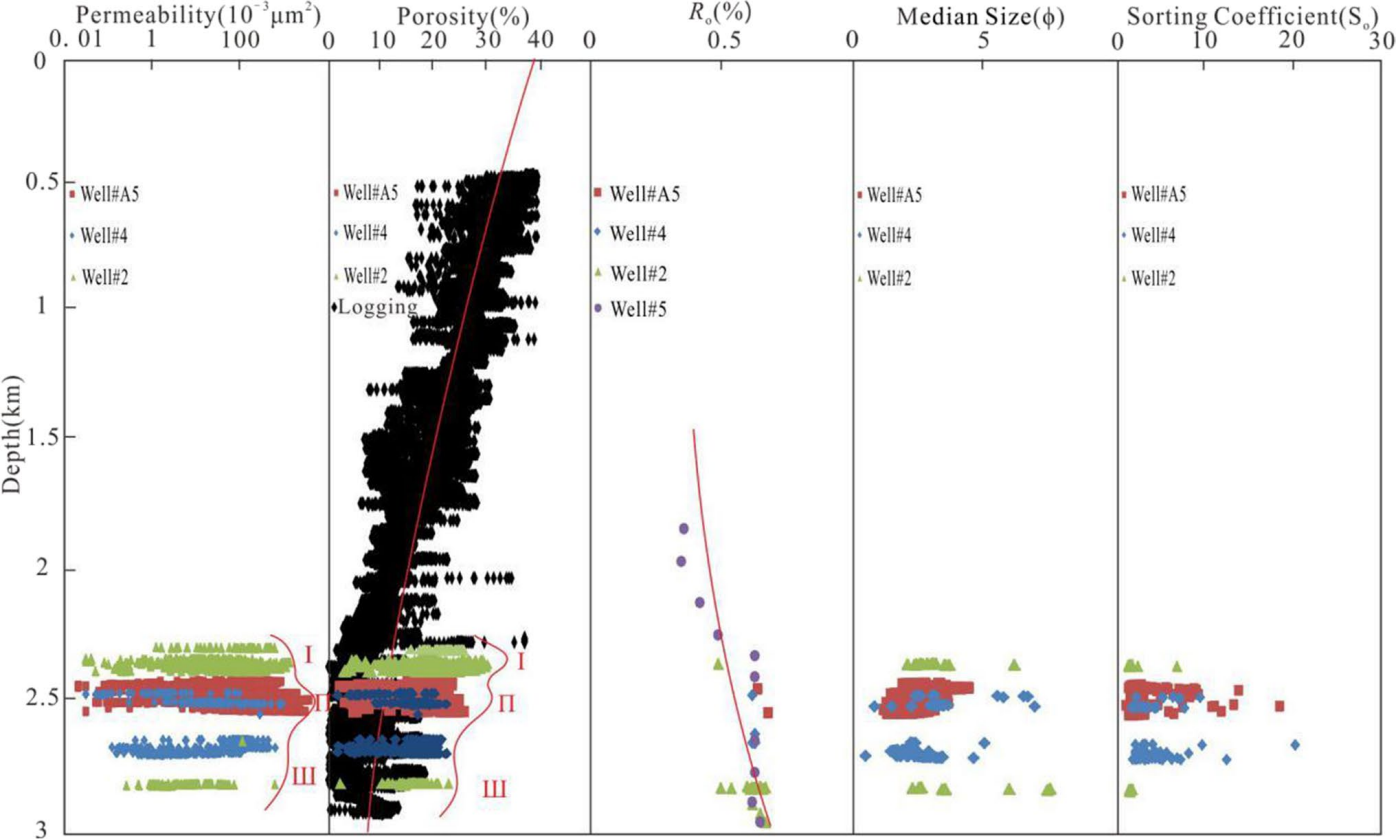
The distribution characteristics of anomalously permeability zones. The red curves indicate the the trend of data points. According to the variation of porosity and permeability, it is further divided into anomalously high porosity and permeability zones I, II, III

The determination and division of anomalously high permeability zones is the premise of the study of anomalously high permeability zones, and also the basis of identifying the sweet spot area under the background of low porosity and low permeability. Based on the porosity depth and permeability depth profiles (Fig. 3) and the compaction simulation curves of Pittman and larese (1991), the distribution range of anomalously high porosity zones in the porosity depth profile is determined. According to the concave convex trend of permeability envelope in permeability depth profile, the distribution range of anomalously high permeability zones are determined.

There are three anomalously high permeability zones in the A_1_^1^, A_1_^2^ and A_2_^1^ micro-stage of the middle diagenetic stage, with the depth range of 2300 m ~ 2400 m, 2400 m ~ 2600 m, 2600 m ~ 2900 m respectively in the E*w*_3_ (Fig. 3, Table 2). The anomalous high permeability zone I is mainly very fine sandstone, with a variable porosity and permeability ranging from 2.40% to 29.70% (mean 19.18%), from 0.03 × 10^−3^ μm^2^ to 1590.00 × 10^−3^ μm^2^ (mean 127.18 × 10^−3^ μm^2^), respectively (Table 2). The anomalous high permeability zone II is mainly fine sandstone, with a variable porosity and permeability ranging from 1.40% to 27.30% (mean 17.35%), from 0.02 × 10^−3^ μm^2^ to 4465.00 × 10^−3^ μm^2^ (mean 297.26 × 10^−3^ μm^2^), respectively (Table 2). The anomalous high permeability zone III is mainly fine sandstone, with a variable porosity and permeability ranging from 1.70% to 30.30% (mean 15.80%), from 0.12 × 10^−3^ μm^2^ to 725.00 × 10^−3^ μm^2^ (mean 61.16 × 10^−3^ μm^2^), respectively (Table 2). It can be seen that the physical property characteristics of anomalously high permeability zoneI, II, III are as follows (Table 2): (1) The average porosity decreases gradually, that is, “high (I) → low (II) → lower (III)” mode; (2) average permeability increases first and then decreases, that is, “low (I) → high (II) → low (III)” mode. Therefore, the porosity and permeability of clastic reservoir are not always positively correlated. In other words, the permeability of the maximum anomalous high porosity zone is not the highest, and the porosity of the maximum anomalous high permeability zone is not necessarily the highest.

**Table 2:** The vertical distribution characteristics of anomalously permeability zones

Anomalously high permeability zones	I	II	III
Depth (m)	2300 m ~ 2400 m	2400 m ~ 2600 m	2600 m ~ 2900 m
Diagenetic period	A_1_^1^	A_1_^2^	A_2_^1^
Main lithology	Very fine sandstone	Fine sandstone	Fine sandstone
Permeability (10^−3^ μm^2^)	$\frac{0.03-1590.00}{127.18\;\left(1366\right)}$	$\frac{0.02-4465.00}{297.26\;\left(1342\right)}$	$\frac{0.12-725.00}{61.16\;\left(344\right)}$
Porosity (%)	$\frac{2.40-29.70}{19.18\;\left(1536\right)}$	$\frac{1.40-27.30}{17.35\;\left(1374\right)}$	$\frac{1.70-30.30}{15.8\;\left(396\right)}$

$$\frac{\mathrm{Minimum}-\mathrm{Maximum}}{\mathrm{Mean}\left(\mathrm{Number}\right)}$$

## The causes of the anomalously high permeability zones

### Clastic grain structure

Sedimentation and diagenesis are the two fundamental factors affecting the physical properties of clastic reservoirs. The characteristics of clastic grain structure are often the embodiment of sedimentation. It can be seen from Tables 2 and 3 that with the increase of burial depth, the anomalous high permeability zone I, II, III have obvious regularity between grain structure and physical properties: (1) The grain size (mm) of clastic particles firstly increases and then decreases, that is “small (I) → big (II)→ small (III)” mode; (2) the sorting ability of clastic particles gradually becomes worse, that is “good (I) → medium (II) → poor (III)” mode. It can be seen that the variation tendency of porosity is consistent with the sorting of clastic particles, and the variation trend of permeability is consistent with the grain size of clastic particles. In other words, the porosity of the reservoir is mainly controlled by the sorting, and the permeability of the reservoir is primarily controlled by the grain size.

**Table 3:** The vertical distribution characteristics of anomalously permeability zones

Anomalously high permeability zones	I	II	III
Depth(m)	2300 m ~ 2400 m	2400 m ~ 2600 m	2600 m ~ 2900 m
Median size/ϕ	$\frac{2.09-6.14}{3.10\;\left(16\right)}$	$\frac{0.85-6.91}{2.67\;\left(259\right)}$	$\frac{0.50-7.50}{2.68\;\left(67\right)}$
Sorting/S_o_	$\frac{1.23-6.82}{1.85\;\left(16\right)}$	$\frac{0.99-18.64}{2.97\;\left(265\right)}$	$\frac{1.30-20.40}{4.00\;\left(67\right)}$

$$\frac{\mathrm{Minimum}-\mathrm{Maximum}}{\mathrm{Mean}\left(\mathrm{Number}\right)}$$

**Fig. 4:**
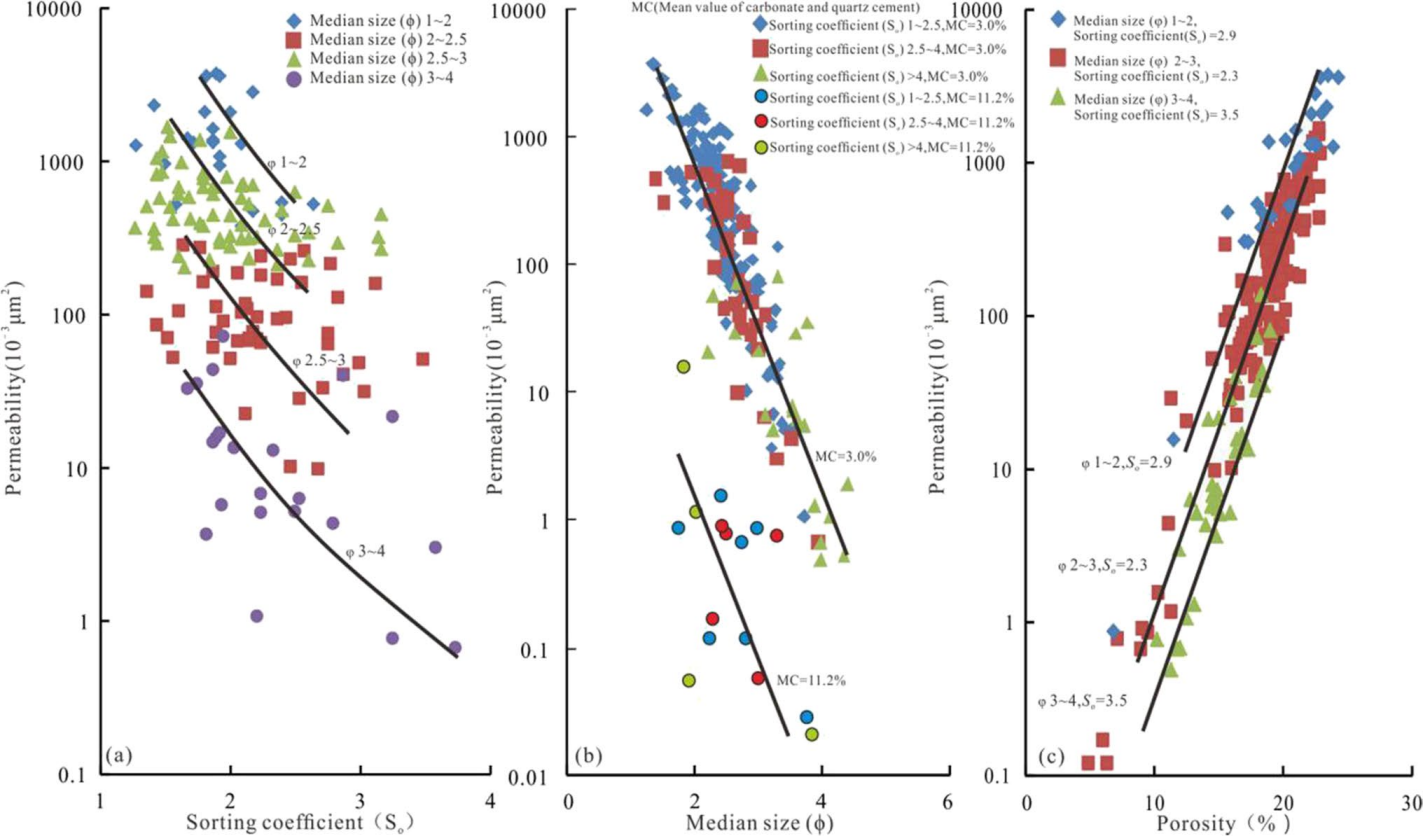
Relationship between porosity, particle size, sorting coefficient and permeability

In order to further study the effect of clastic particles on reservoir permeability, the relationship between grain size sorting coefficient porosity and permeability is analyzed (Fig. 4). The results show that, when the grain size of clastic particles were 1 ~ 2, 2 ~ 2.5, 2.5 ~ 3 and 3 ~ 4 (Fig. 4a), the permeability decreased with the increase of sorting coefficient between 10,000 × 10^−3^ μm^2^ ~ 1000 × 10^−3^ μm^2^, 1000 × 10^−3^ μm^2^ ~ 100 × 10^−3^ μm^2^, 100 × 10^−3^ μm^2^ ~ 10 × 10^−3^ μm^2^, 10 × 10^−3^ μm^2^ ~ 0.1 × 10^−3^ μm^2^ respectively. When the sorting is 1 ~ 2.5, 2.5 ~ 4 (Fig. 4b), the trend of permeability decrease with the increase of the median grain size (ϕ) is more concentrated, the range of permeability variation is mainly concentrated between 1000 × 10^−3^ μm^2^ ~ 1 × 10^−3^ μm^2^. When the cement content is high (mean 11.2%) (Fig. 4b), the permeability is significantly lower. Therefore, the relationship between permeability and sorting is controlled by grain size. The relationship between permeability and grain size is controlled by cement content. Figure 4c shows that, the permeability and porosity of reservoir are controlled by grain size and sorting. When the particle size and sorting are 1 ~ 2 and 2.9, respectively (Fig. 4c), the permeability or porosity is higher than that when the particle size and sorting are 2 ~ 3 and 2.3 respectively, under the same conditions (i.e., when the permeability or porosity is constant). Therefore, the effect of grain size on permeability is stronger than that of sorting, and the effect of sorting on porosity is stronger than that of grain size. The anomalously high permeability zone I, II are considered to be the strong proof that the effects of grain size and sorting on permeability and porosity are different. It can be seen from Fig. 3, Tables 2 and 3 that the anomalous high permeability zone I with the smallest grain size and well sorted, has the highest porosity compared with the anomalous high permeability zone II and III. The anomalous high permeability zone II with the biggest grain size and moderately sorted, has the highest permeability compared with the anomalous high permeability zone I and III.

In generally, the grain size and sorting of clastic particles have an important influence on reservoir porosity and permeability. The control effect of the grain size on permeability is stronger than that of sorting, and the control effect of sorting on porosity is stronger than that of grain size. This is also an important reason for the obvious inconsistency about variation of permeability and porosity in the anomalous high permeability zone I, II, III. Therefore, when the grain size is coarse, it is easier to form anomalous high permeability zones (the anomalous high permeability zone I); when the sorting is good, it is easier to form anomalous high porosity zones.

### Dissolution

**Fig. 5:**
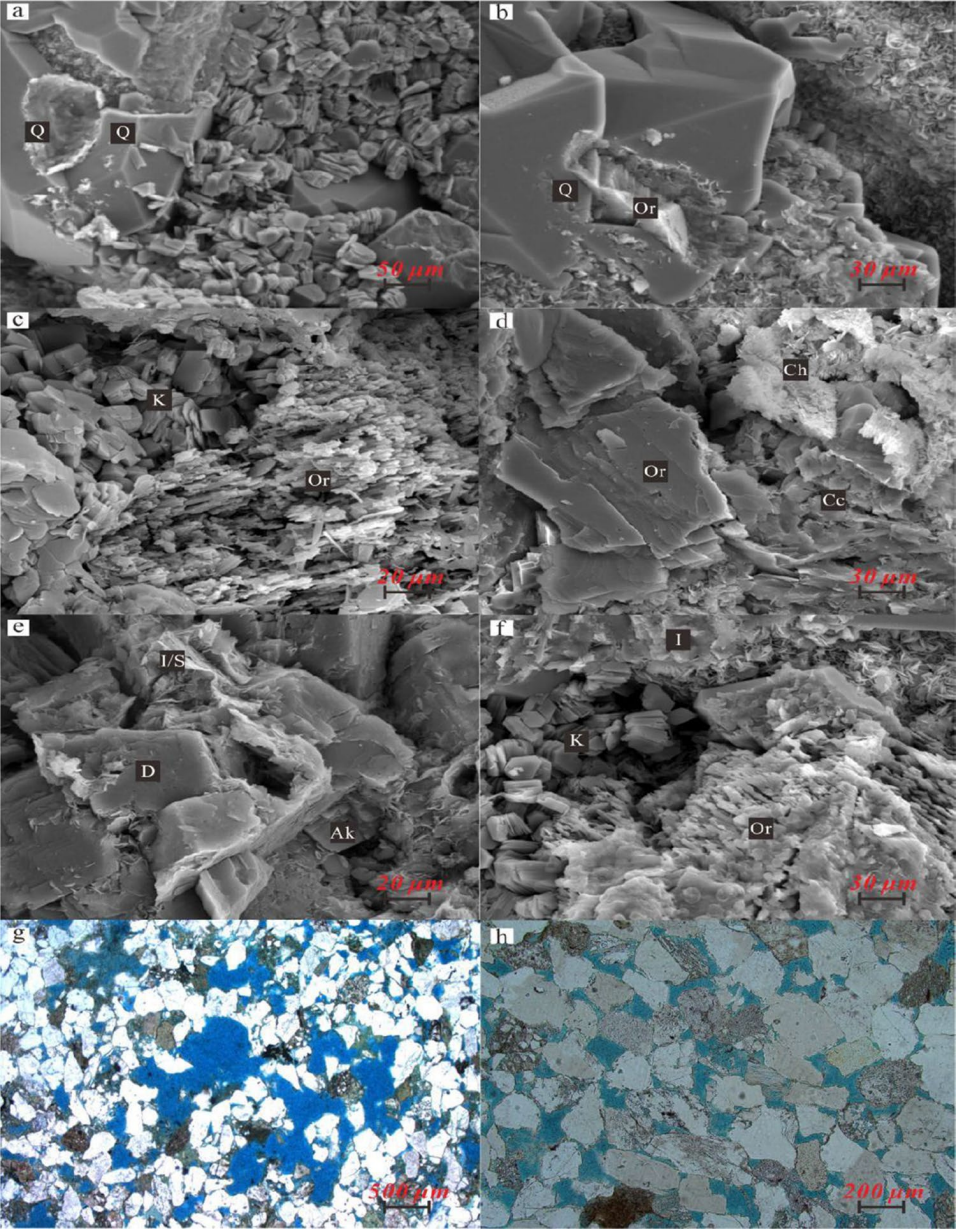
Dissolution characteristics of sandstone reservoir in the E*w*_3_. Quartz (Q) was dissolved, Well#B33, (SEM), 2468.30 m, ×700. **b** Quartz (Q) was dissolved. Potassium feldspar (Or) is filled in quartz caves. Well#B33, SEM, 2474.60 m, ×2000. **c** Potassium feldspar (Or) was dissolved and concomitant precipitation of kaolinite (K), Well#B33, SEM, 2948.78 m, ×2000. **d** Calcite (Cc) was dissolved. Calcite (Cc) and chlorite (Ch) were deposited between grains. Well#B33, SEM, 2482.71 m, ×1500. **e** Dolomite (D) and ankerite (Ak) were dissolved. Dolomite (D), ankerite (Ak) and I/S mixed layer were deposited between grains. Well#B33, SEM, 2471.82 m, ×2500. **f** Illite (I) and Potassium feldspar (Or) were dissolved. Kaolinite (K) and Illite (I) were deposited between grains. Well#B33, SEM, 2942.34 m, ×1500. **g** Potassium feldspar (Or) was dissolved and concomitant secondary porosity increase. Well#A5, (MSI), 2817.84 m, ×4(−). **h** Lithic fragment was dissolved and concomitant secondary porosity increase. Well#A5, MSI, 2736.16 m, ×50(−)

**Fig. 6:**
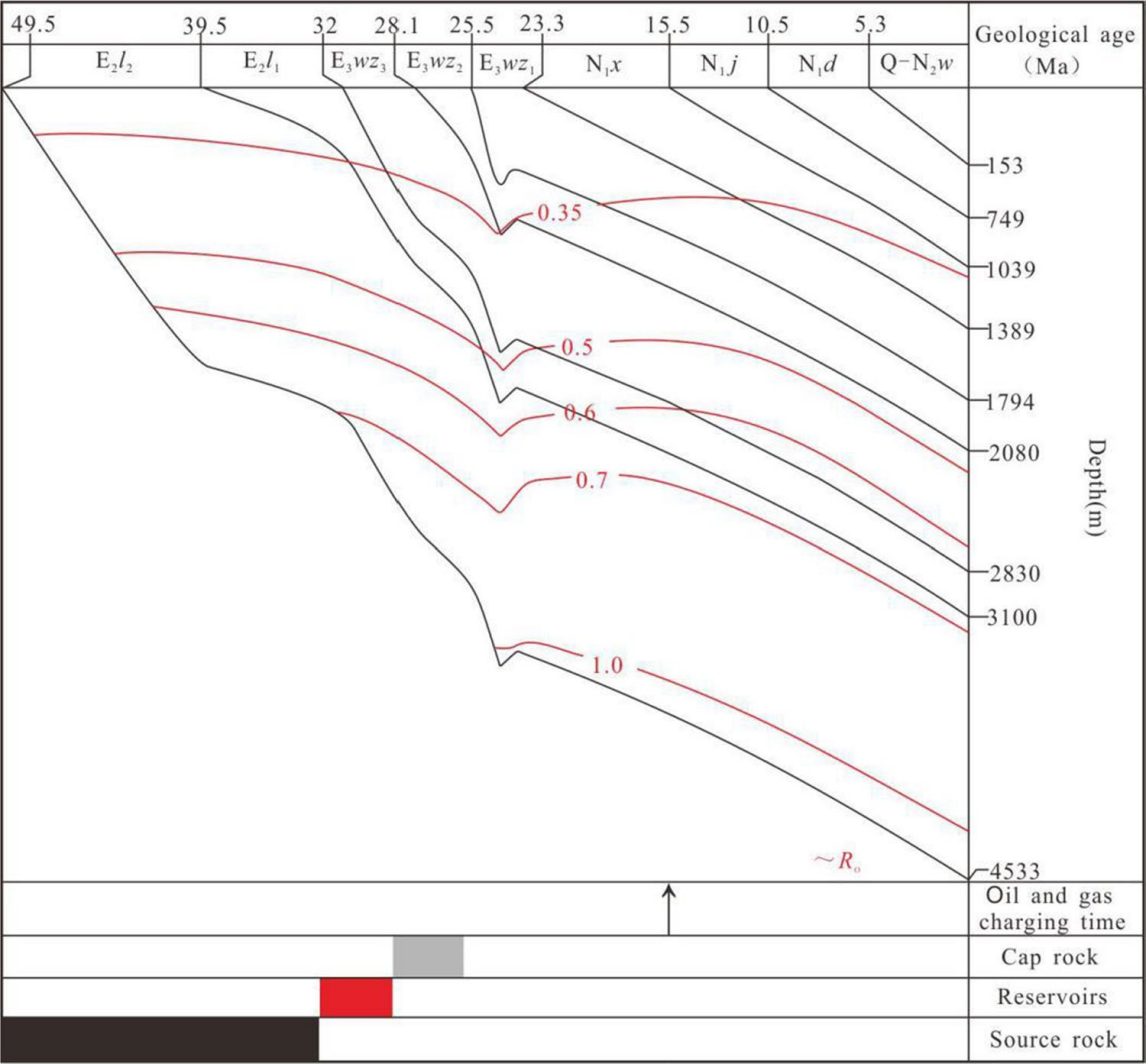
Comprehensive figure of burial history, Well#2,in the Weizhou 12-X oilfield, Weixinan Sag

**Fig. 7:**
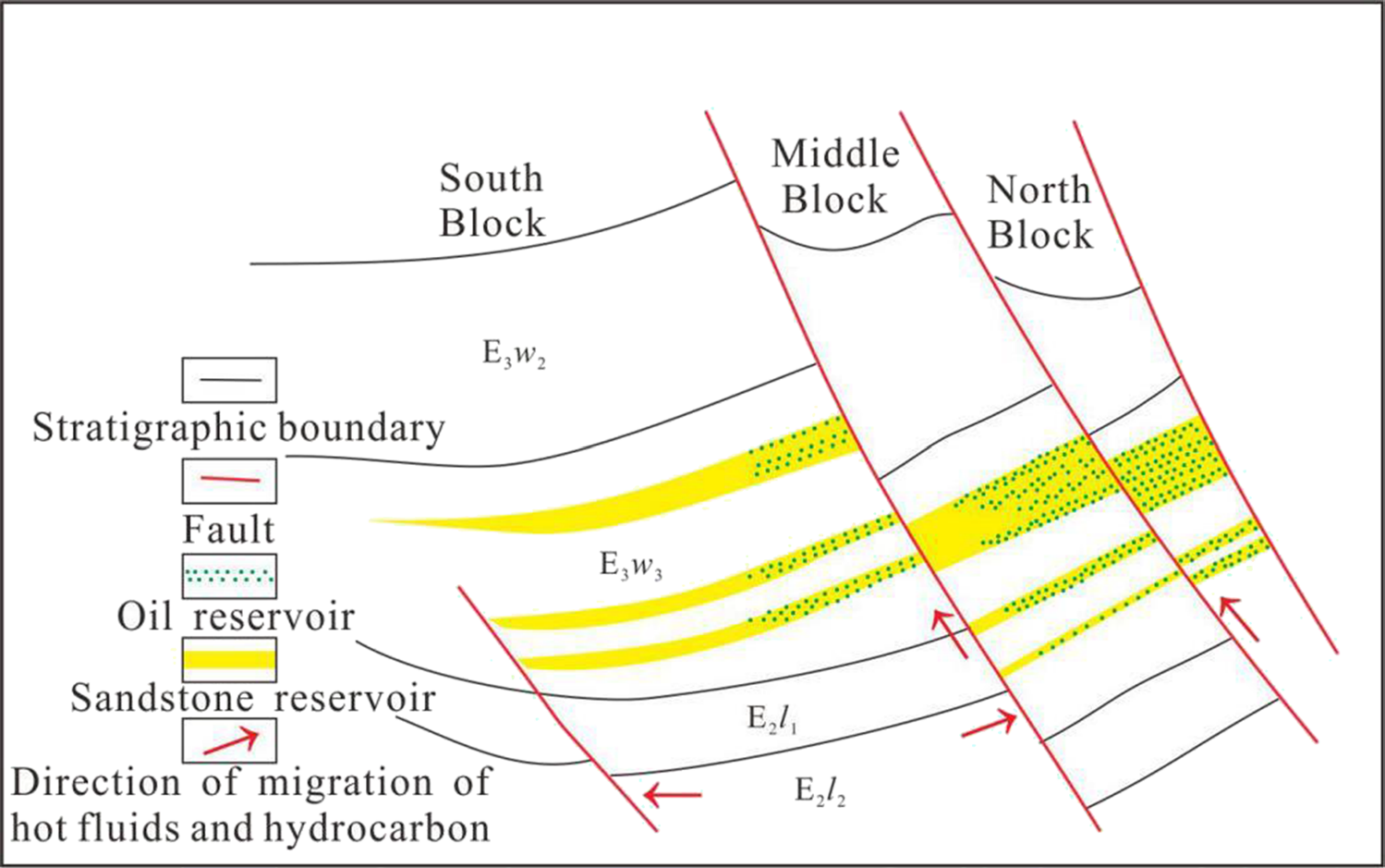
Schematic diagram of hydrocarbon migration path in the study area

In this paper, the geological information of hydrothermal fluid in Weizhou formation is revealed by means of MSI, SEM and diagenetic numerical simulation software system (Fig. 5). The burial history and thermal evolution history of organic matter in well#2 of Weizhou 12-X oilfield is also recovered (Fig. 6). The results show that, there are two important periods in the diagenetic evolution process of Weizhou Formation: (1) From the early Paleocene to the end of Oligocene (Guo et al. 2010), with intense tectonic activity, the magmatic hydrothermal fluid was transported to the sandstone reservoir of Weizhou Formation by the large faults (Fig. 7). (2) From the middle late period of Weizhou Formation deposition to the sedimentary period of Xiayang Formation (Gan et al. 2017), the large scale tectonic uplift and denudation occurred in the study area, resulting in an increase of pressure between source and reservoir, accompanied by the large scale migration of oil and gas in the early stage. And then, oil and gas entered the sandstone reservoir in the E*w*_3_ along the faults (Fig. 7).

**Fig. 8:**
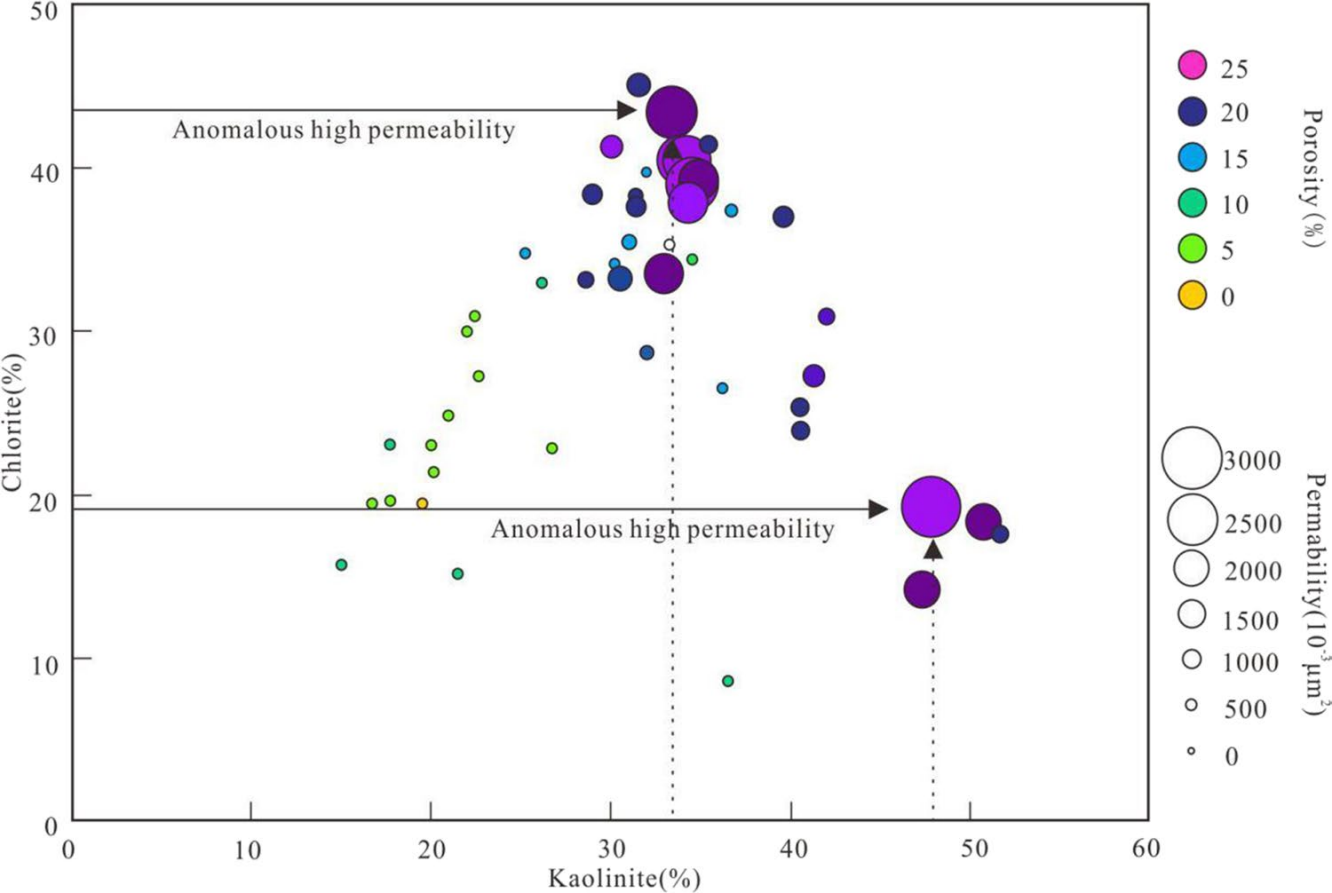
Relationship between kaolinite and chlorite in the E*w*_3_,Well#A5,Weizhou 12-X oilfield. The circle size is directly proportional to the permeability, and the color variation is closely related to the porosity

**Fig. 9:**
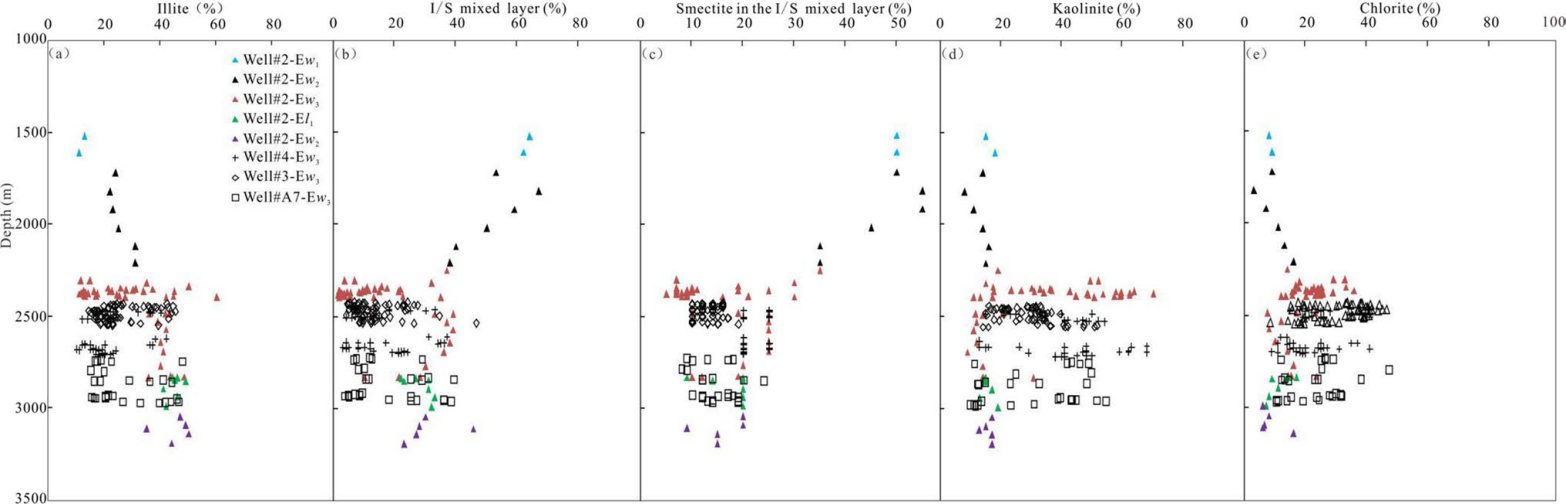
Variation of different types of clay with burial depth in Weixinan Sag

Our further microscopic observation and diagenetic numerical simulation results show that, (1) From the beginning of Paleocene to the end of Oligocene, Hydrothermal alkali metasomatism (Du 1986; Qiu et al. 2001) occurred between the deep magmatic hydrothermal solution and the rocks in the E*w*_3_, which eroded quartz (Fig. 5a, b) and produced secondary pores. At the same time, a large amount of Fe^2+^,Mg^2+^ are concentrated in the E*w*_3_, which promoted the formation of authigenic chlorite. As a result, the position of anomalous high authigenic chlorite content is consistent with that of anomalous high permeability zones (Figs. 3, 8 and 9). Figure 8 shows that, when authigenic chlorite content is as high as 43.60%, the permeability is 2319.00 × 10^−3^ μm^2^. (2) From the middle late stage of Weizhou Formation deposition to the sedimentary period of Xiayang Formation, the *R*_o_ of the source rocks in the E*l*_2_ is mainly in the range from 0.7% to 1.0%, which is in the mature stage of massive hydrocarbon generation. The source rocks at the bottom of E*l*_2_ are close to the peak of oil generation (*R*_o_ = 1.0%) (Fig. 6). Organic acids and CO_2_, as the products of oil generation from organic matter, dissolved in water to form an acidic hot fluid. The acidic fluid was transported to reservoirs in the E*w*_3_. The unstable minerals in the reservoirs, such as feldspar, carbonate cement, debris, clay minerals, are dissolved (Fig. 5c–f), accompanied by the generation of secondary pores (Fig. 5g, h). As a result, the position of anomalous high authigenic kaolinite content is consistent with that of anomalous high permeability zones (Figs. 3, 8 and 9). Figure 8 shows that, when authigenic kaolinite content is as high as 47.20%, the permeability is 2536.00 × 10^−3^ μm^2^.

As can be seen from Figs. 3 and 9, vertically, the abnormal transformation sites of clay minerals are consistent with that of the anomalous high permeability zones, which both located between 2300 m and 2900 m. Therefore, the abnormal variation of clay mineral content can be considered as a typical sign of the formation of anomalous high permeability zones. It is also a powerful proof that dissolution is considered to be one of the causes of anomalous high permeability zones. And furthermore, authigenic kaolinite and chlorite can be regarded as the indicator minerals for the formation of the anomalous high permeability zones in acidic and alkaline diagenetic environments respectively.

### Hydrocarbon emplacement

The burial history and thermal evolution history of organic matter in well#2 of Weizhou 12-X oilfield is recovered, as shown in Fig. 6. During hydrocarbon emplacement at the end of Xiayang Formation deposition (15.5 Ma), the source rocks in the E*l*_2_ are in the mature stage of hydrocarbon generation, with the variation of *R*_o_ between 0.7% and 1.0%. *R*_o_ of sandstone reservoir in the E*w*_3_ is between 0.35% and 0.6%. The relationship between *R*_o_ of source rocks in the E*l*_2_ and *R*_o_ of sandstone reservoir in the E*w*_3_ is as follows:

1 $${R}_o^s>{R}_o^r$$

where *R*_o_^s^ is the *R*_o_ of source rocks in the E*l*_2_, *R*_o_^r^ is the *R*_o_ of sandstone reservoir in the E*w*_3_.

E*w*_3_ with still in the early diagenetic stage, mainly develops early cementation diagenetic facies and early dissolution diagenetic facies. Therefore, the charging of oil and gas is the early emplacement.

The diagenetic reaction system in the E*w*_3_ was influenced by early emplacement of hydrocarbons, which changed from water - rock diagenetic system to water - oil - rock diagenetic system. As a result, the oil saturation increases, the water saturation decreases and the cementation weakens. In addition, the porosity and permeability decrease, and the reservoir space and infiltration channels are well protected.

**Fig. 10:**
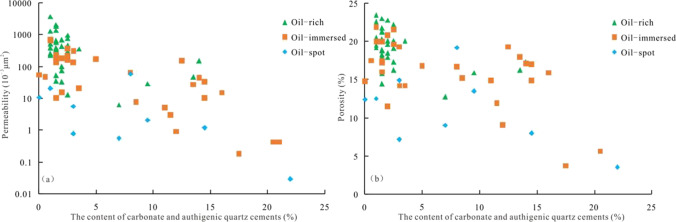
The relationship between oiliness, carbonate and quartz cements, and reservoir physical property in the E*w*_3_

The statistical results of oil grade, cement content (carbonate cement and authigenic quartz), porosity and permeability of sandstone samples in the E*w*_3_ further support the above viewpoint, as shown in Fig. 10 and Table 4. (1) When the sandstone sample is oil-rich (Table 4), the content of carbonate and authigenic quartz cements is low, with an average of 2.74%, and the reservoir permeability and porosity are high (Fig. 10a, b, Table 4). The number of samples with permeability greater than 50.00 × 10^−3^ μm^2^ and porosity greater than 15.00% accounts for 86.00% and 94.00%, respectively. (2) When the oil grade of sandstone samples is oil-immersed, the content of carbonate and authigenic quartz cements is higher, with an average of 7.40%, and the reservoir permeability and porosity are relatively low (Fig. 10a, b, Table 4). The proportion of samples with permeability greater than 50.00 × 10^−3^ μm^2^ and porosity greater than 15.00% is 44.00% and 66.00%, respectively. (3) When the oil grade of sandstone samples is oil spot, the content of carbonate and authigenic quartz cements is the highest, with an average value of 7.60%, and the reservoir permeability and porosity are the lowest (Fig. 10a, b, Table 4). The number of samples with permeability greater than 50.00 × 10^−3^ μm^2^ and porosity greater than 15.00% accounts for only 11.00% and 11.00%, respectively. Therefore, early emplacement of hydrocarbons in the E*w*_3_ can occupy the reservoir space early, which largely inhibits the progress of cementation and alleviates the damage of cementation to the porosity and permeability. It is beneficial to the preservation of porosity and permeability in the E*w*_3_.

**Table 4:** Statistical table of oil-bearing grade of core, porosity, permeability,carbonate and quartz cements in the Weizhou formation,12-X field, Weixinan Sag

Oil-bearing grade of core	cement content (carbonate cement and authigenic quartz)	Porosity (%)	Permeability (10^−3^ μm^2^)
Oil-rich	$\frac{1.00-14.00}{2.74\left(36\right)}$	$\frac{12.80-23.50}{19.30\left(36\right)}$	$\frac{6.30-3716.00}{592.70\left(36\right)}$
Oil-immersed	$\frac{0.03-21.30}{7.40\left(36\right)}$	$\frac{3.70-21.90}{15.70\left(31\right)}$	$\frac{0.20-725.00}{129.20\left(31\right)}$
Oil-spot	$\frac{0.03-22.00}{7.60\left(9\right)}$	$\frac{3.50-19.20}{11.10\left(9\right)}$	$\frac{0.03-58.20}{11.10\left(9\right)}$

## Discussion

The clastic grain structure not only controls the original physical properties of the reservoir, but also has an important effect on the evolution of porosity and permeability during diagenesis. The mechanical compaction experiment shows that the coarse-grained sand is more compressible due to more grain fracturing than in the fine-grained sand (Chuhan et al. 2002, 2003). Then, the pore structure of coarse sandstone is more severely damaged due to more fracturing than in fine-grained sandstone. Subsequently, the original reservoir permeability of the coarse-grained sandstone is lost even more due to mechanical compaction. However, within a limited range (i.e., the fine-grained sandstone), the greater the grain size, the lower the permeability reduction may be. Poorly sorted sands have much lower initial porosity than well sorted sands, but show less porosity loss by mechanical compaction (Fawad et al. 2011).

In addition, fine-grained sandstones developed in delta facies are more likely to form anomalously high permeability zones, especially delta distributary channel subfacies. The main reason is closely related to the precipitation of chlorite coating in the early stage of diagenesis. Delta is the main environment for authigenic chlorite coating, and the number of research examples of the positive correlation between chlorite coating and reservoir quality accounts for the majority (Dowey et al. 2012; Chen et al. 2021). The results of microscopic observation of existing coring Wells in this study area show that the occurrence of chlorite in the reservoir is mainly coating or lining, except Well B33 (Chen et al. 2021). Furthermore, Coated or lined chlorite is always positively correlated with reservoir permeability, while limiting the factors that control reservoir quality (Chen et al. 2021). This is why the permeability is as high as 2319.00 × 10^−3^ μm^2^ even when the chlorite content is 43.60% (Fig. 8). As shown in Fig. 9, the mean (39.22%) and maximum (70.10%) kaolinite content of the anomalously high permeability zone I is significantly higher than mean (30.07%) and maximum (51.60%) in the anomalously high permeability zone II, the mean (22.00%) and maximum (35.70%) chlorite content of the anomalously high permeability zone I is obviously lower than mean (30.40%) and maximum (46.40%) in the anomalously high permeability zone II. As shown in Fig. 3 and Table 1, the mean (19.18%) and maximum (29.70%) porosity of the anomalously high permeability zone I is higher than mean (17.35%) and maximum (27.30%) in the anomalously high permeability zone II,the mean (297.26 × 10^−3^ μm^2^) and maximum (4465.00 × 10^−3^ μm^2^) permeability of the anomalously high permeability zone II is visibly higher than mean (127.18 × 10^−3^ μm^2^) and maximum (1590.00 × 10^−3^ μm^2^) in the anomalously high permeability zone I. Therefore, the precipitation of authigenic chlorite may be more conducive to improving the permeability than the porosity; the precipitation of authigenic kaolinite may be more conducive to increasing secondary porosity than to improving permeability. And further, The precipitation of both authigenic chlorite and kaolinite occurred during the period of thermal fluid activity, which occurred from the Early Paleocene to the end of Oligocene and from the Middle Late Period of Weizhou Formation to the sedimentary period of Xiayang Formation, respectively. In other words, the best diagenetic stage conducive to the formation of anomalously high permeability zone is the stage of hot fluid activity. At present, the anomalously high permeability zones is in the middle diagenetic stage from A_1_^1^ to A_2_^1^ micro-stage, and the A_1_^2^ micro-stage is the best. Although the mean (36.90%) and maximum (68.00%) porosity in the anomalously high permeability zone III is higher than in the anomalously high permeability zone II, there is no similar relationship in porosity between anomalously high permeability zone III and II. Because the porosity is also affected by other factors (sorting, etc), that is, compared with other anomalously high permeability zones, the poor porosity of the anomalously high permeability zone III is mainly controlled by poor sorting.

Finally, the early hydrocarbon emplacement is an important geological event to preserve porosity and permeability. This is because higher saturation may have a limiting effect on the water-rock reaction, or even stop a certain type of diagenetic reaction. If there is no key geological event (Hydrocarbon emplacement, Grain coating, etc.) to protect the reservoir porosity or permeability, reservoirs with high porosity and permeability may be destroyed at any time during diagenesis. In general, the formation and preservation of the anomalously permeability zones mostly depend on the effective coupling of multiple factors in different geological periods, while the destruction of the anomalously permeability zones may only need one unremarkable geological factor at any time.

## Conclusions

Combining experimental analysis, microscopic observation and numerical simulation of diagenesis, the effect of grain size, sorting, dissolution, and early emplacement of hydrocarbons on the formation, distribution and origin of anomalously high permeability zones are discussed. The preliminary conclusions are as follows:

1. The characteristics of porosity and permeability in the E*w*_3_ are controlled by grain size and sorting. Grain size and sorting have different controlling effects on porosity and permeability: 1) The sorting has a stronger effect on porosity than grain size, and the anomalously high permeability zone I, III with the highest, lowest porosity in the E*w*_3_ are two typical examples respectively. 2) The grain size has a stronger effect on permeability than sorting, and the anomalously high permeability zone II with the highest permeability in the E*w*_3_ is a typical example.
2. The magmatic hydrothermal fluid and organic acids entered the reservoir of the E*w*_3_ in different periods, and dissolved unstable minerals such as quartz and feldspar respectively, forming secondary pores. At the same time, with the abnormal transformation of authigenic chlorite and kaolinite, authigenic chlorite and kaolinite can be used as the indicator minerals for the anomalously high permeability zones with different origins in alkaline and acid media, respectively. The main reason is that the sites of abnormal transformation of clay minerals is consistent with the distribution of anomalously high permeability zones in the E*w*_3_.
3. Significant porosity and permeability preservation is associated with early emplacement of hydrocarbons. Because early emplacement of hydrocarbons not only retard cementation but also prevent the decrease in porosity and permeability due to precipitation of cements.
